# Supplementary material for: Inhibition of SARS-CoV-2 (previously 2019-nCoV) infection by a highly potent pan-coronavirus fusion inhibitor targeting its spike protein that harbors a high capacity to mediate membrane fusion
Source: Cell Res. 2020 Mar 30;30(4):343–55. doi: 10.1038/s41422-020-0305-x (PMC7104723; doi:10.1038/s41422-020-0305-x)
Supplement: Supplementary file 1 — Supplementary information, Fig. S1 [file 41422_2020_305_MOESM1_ESM.pdf]

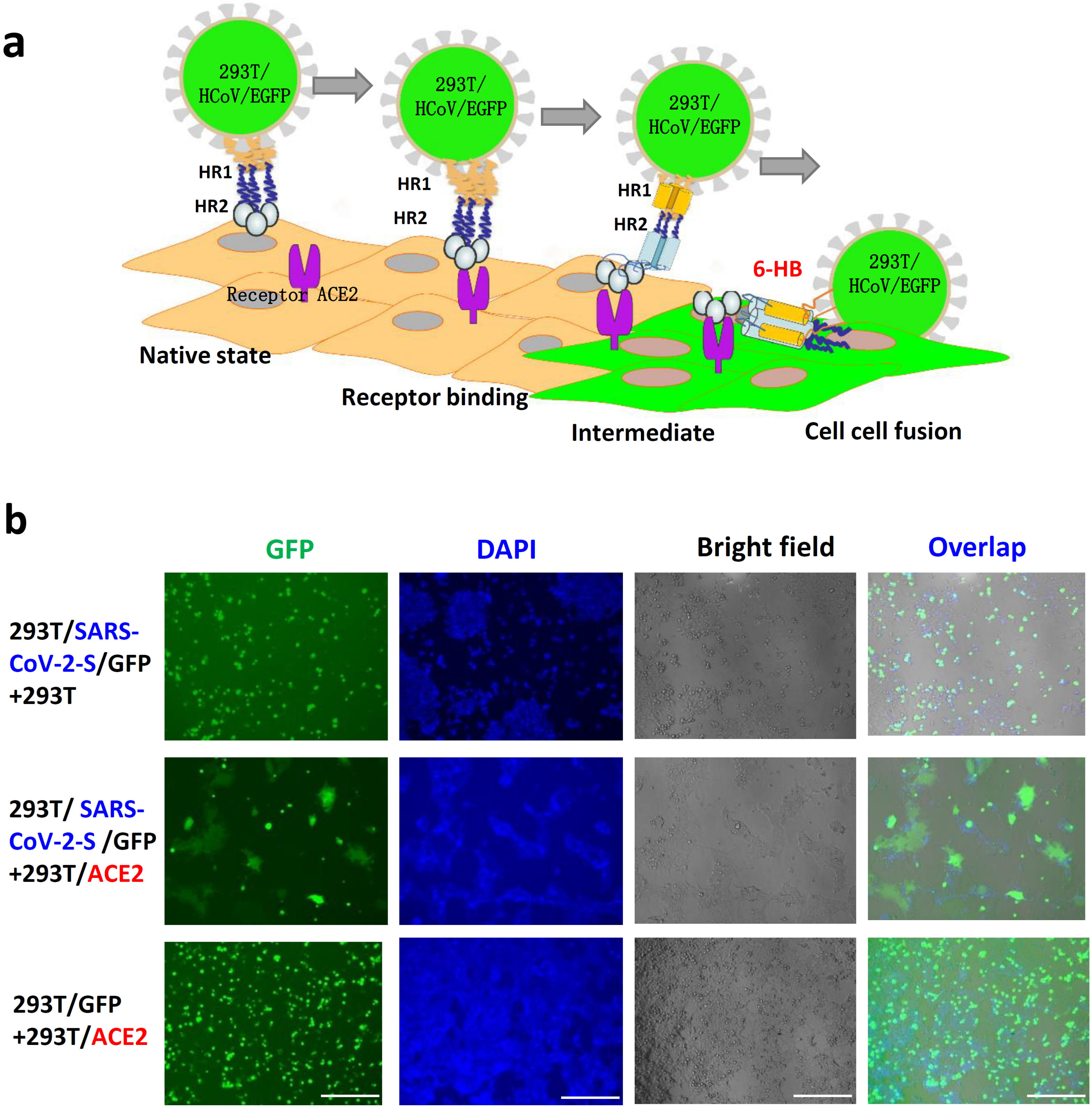
a
293T/
HCoV/EGFP
HR1
HR2
Receptor ACE2
6-HB
Native state
Receptor binding
Intermediate
Cell cell fusion
b
GFP
DAPI
Bright field
Overlap
293T/SARS-CoV-2-S/GFP +293T
293T/ SARS-CoV-2-S /GFP +293T/ACE2
293T/GFP +293T/ACE2

Supplementary information, Fig. S1 Establishment of SARS-CoV-2 S-mediated cell-cell fusion.

a. Schematic representation of SARS-CoV-2 S-mediated cell-cell fusion. **b**. Images of cell-cell fusion between 293T/SARS-CoV-2/EGFP cells and 293T cells (upper), 293T/SARS-CoV-2/EGFP cells and 293T/ACE2 cells (middle), 293T/EGFP cells and 293T/ACE2 cells (lower). Scale bar = 400 μm.
