# Supplementary material for: Inhibition of SARS-CoV-2 (previously 2019-nCoV) infection by a highly potent pan-coronavirus fusion inhibitor targeting its spike protein that harbors a high capacity to mediate membrane fusion
Source: Cell Res. 2020 Mar 30;30(4):343–55. doi: 10.1038/s41422-020-0305-x (PMC7104723; doi:10.1038/s41422-020-0305-x)
Supplement: Supplementary file 2 — Supplementary information, Fig. S2 [file 41422_2020_305_MOESM2_ESM.pdf]

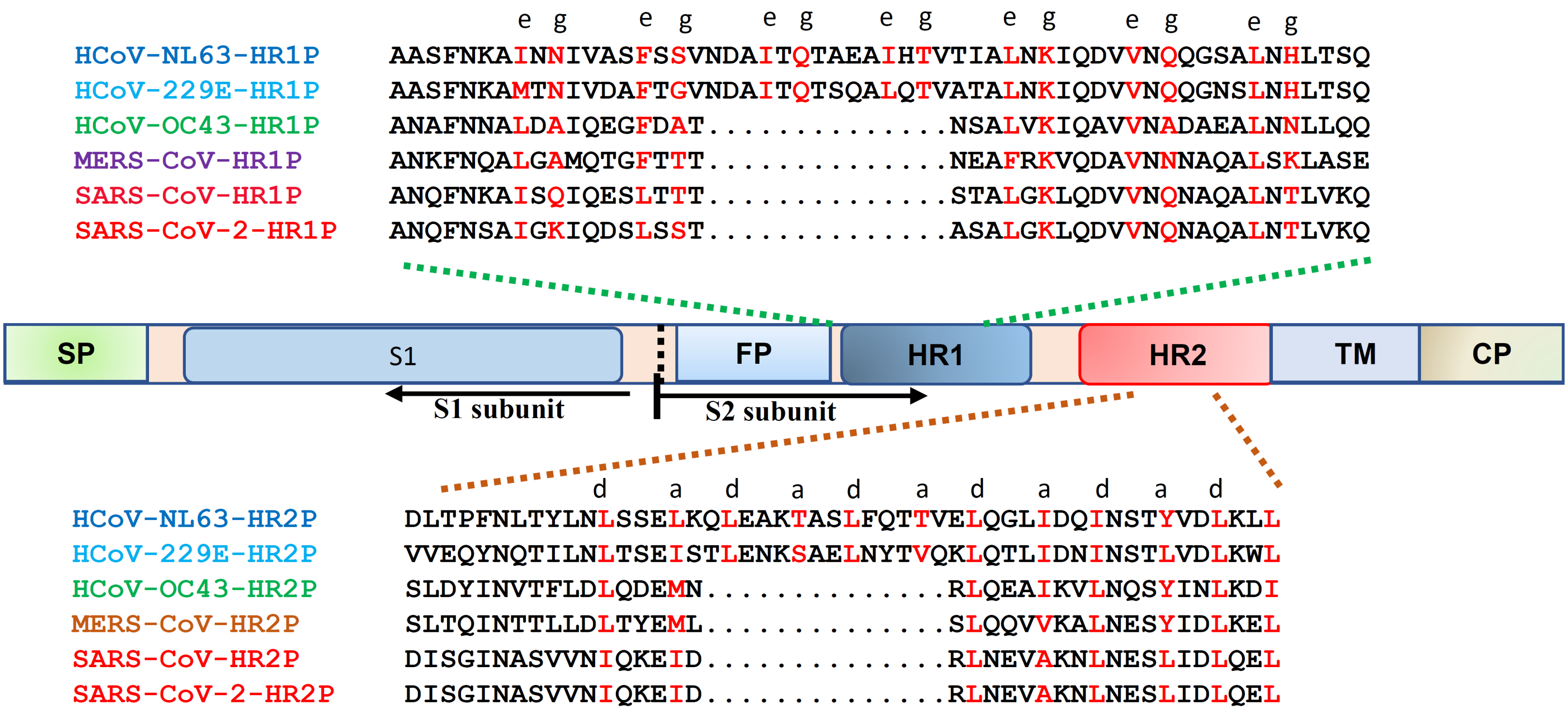

e g e g e g e g e g e g e g
HCoV-NL63-HR1P AASFNKAINNIVASFSSVNDAITQTAEAIHTVTIALNKIQDVVNQQGSALNHLTSQ
HCoV-229E-HR1P AASFNKAMTNIVDAFTGVNDAITQTSQALQTVATALNKIQDVVNQQGNSLNHLTSQ
HCoV-OC43-HR1P ANAFNNALDAIQEGFDAT..............NSALVKIQAVVNADAEALNNLLQQ
MERS-CoV-HR1P ANKFNQALGAMQTGFTTT..............NEAFRKVQDAVNNNAQALSKLASE
SARS-CoV-HR1P ANQFNKAISQIQESLTTT..............STALGKLQDVVNQNAQALNTLVKQ
SARS-CoV-2-HR1P ANQFNSAIGKIQDSLSST..............ASALGKLQDVVNQNAQALNTLVKQ
SP
S1
FP
HR1
HR2
TM
CP
S1 subunit
S2 subunit
d a d a d a d a d a d
HCoV-NL63-HR2P DLTPFNLTYLNLSSELKQLEAKTASLFQTTVELQGLIDQINSTYVDLKLL
HCoV-229E-HR2P VVEQYNQTILNLTSEISTLENKSAELNYTVQKLQTLIDNINSTLVDLKWL
HCoV-OC43-HR2P SLDYINVTFLDLQDEMN..............RLQEAIKVLNQSYINLKDI
MERS-CoV-HR2P SLTQINTTLLDLTYEML..............SLQQVVKALNESYIDLKEL
SARS-CoV-HR2P DISGINASVVNIQKEID..............RLNEVAKNLNESLIDLQEL
SARS-CoV-2-HR2P DISGINASVVNIQKEID..............RLNEVAKNLNESLIDLQEL

Supplementary information, Fig. S2 Schematic representation of HCoV S protein and the sequences of the designed peptides (HR1Ps and HR2Ps).
