# Supplementary figures and images for: Inhibition of SARS-CoV-2 (previously 2019-nCoV) infection by a highly potent pan-coronavirus fusion inhibitor targeting its spike protein that harbors a high capacity to mediate membrane fusion
Source: Cell Res. 2020 Mar 30;30(4):343–55. doi: 10.1038/s41422-020-0305-x (PMC7104723; doi:10.1038/s41422-020-0305-x)

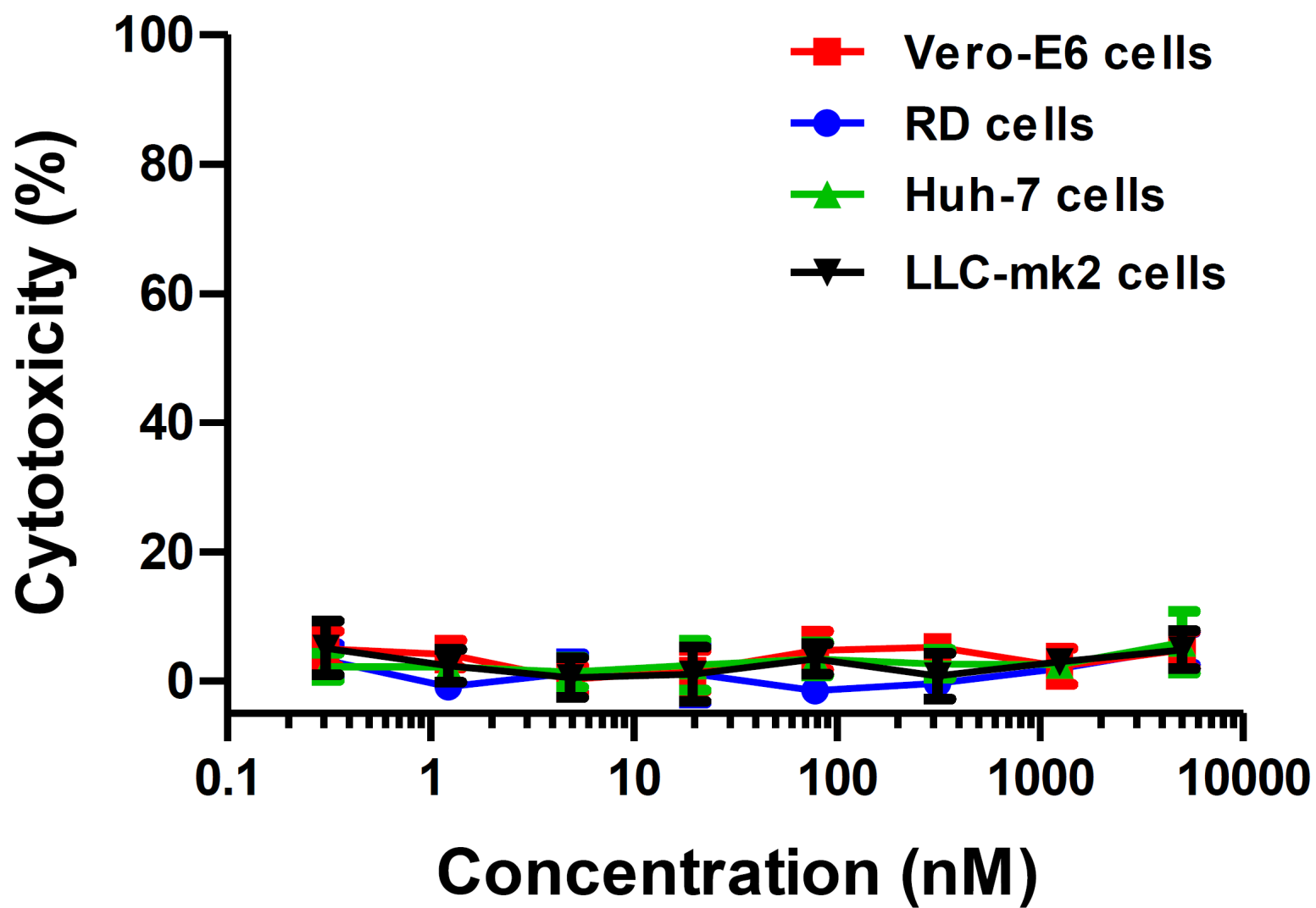

**Supplementary information, Fig. S3 Cytotoxicity of EK1C4 on Vero-E6, RD, Huh-7 and LLC-MK2 cells.**

Supplement: Supplementary file 3 — Supplementary information, Fig. S3 [file 41422_2020_305_MOESM3_ESM.pdf]
