# Supplementary material for: Inhibition of SARS-CoV-2 (previously 2019-nCoV) infection by a highly potent pan-coronavirus fusion inhibitor targeting its spike protein that harbors a high capacity to mediate membrane fusion
Source: Cell Res. 2020 Mar 30;30(4):343–55. doi: 10.1038/s41422-020-0305-x (PMC7104723; doi:10.1038/s41422-020-0305-x)
Supplement: Supplementary file 4 — Supplementary information, Fig. S4 [file 41422_2020_305_MOESM4_ESM.pdf]

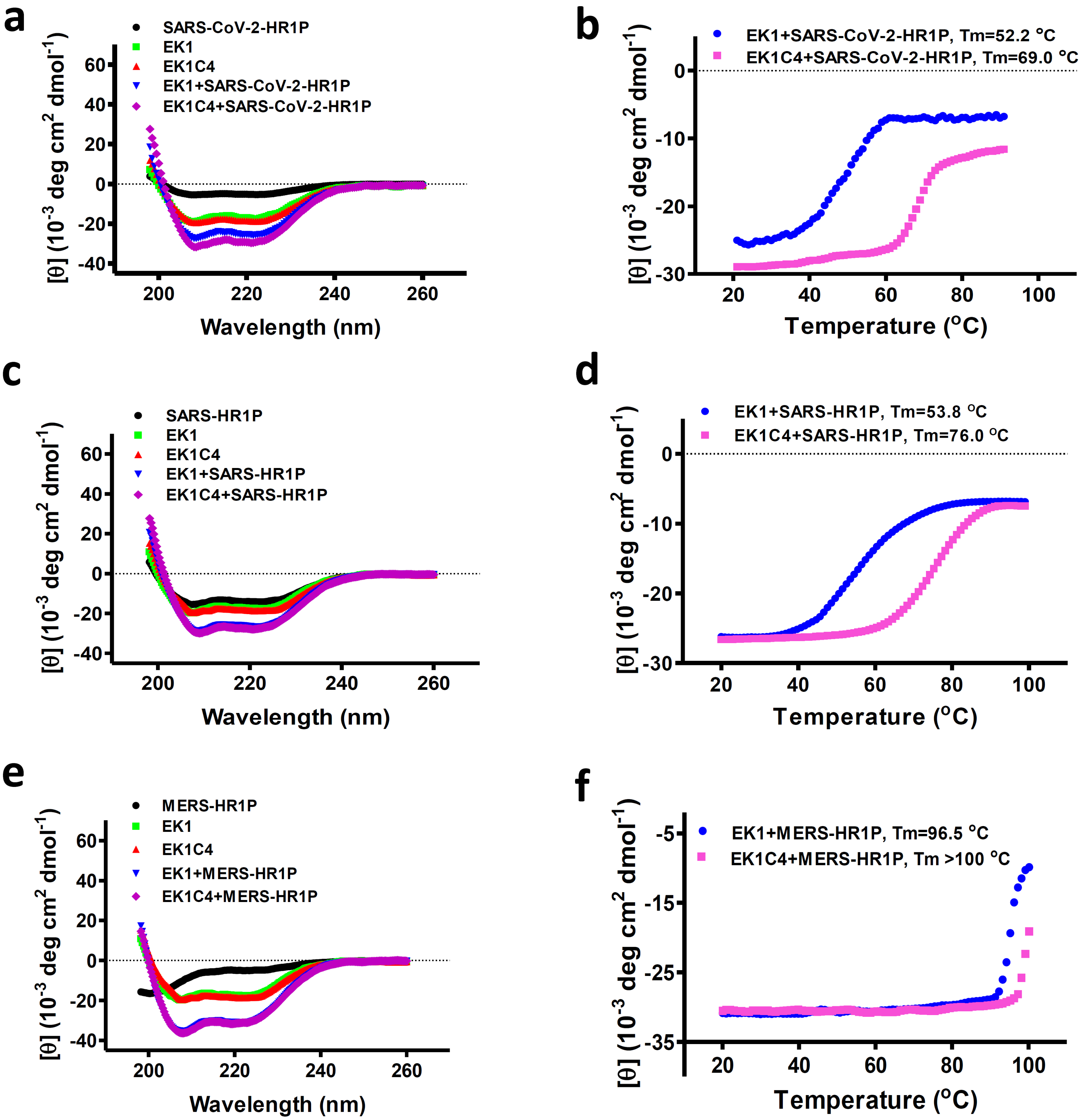
a
SARS-CoV-2-HR1P
EK1
EK1C4
EK1+SARS-CoV-2-HR1P
EK1C4+SARS-CoV-2-HR1P
[θ] (10-3 deg cm2 dmol-1)
Wavelength (nm)
b
EK1+SARS-CoV-2-HR1P, Tm=52.2 °C
EK1C4+SARS-CoV-2-HR1P, Tm=69.0 °C
[θ] (10-3 deg cm2 dmol-1)
Temperature (°C)
c
SARS-HR1P
EK1
EK1C4
EK1+SARS-HR1P
EK1C4+SARS-HR1P
[θ] (10-3 deg cm2 dmol-1)
Wavelength (nm)
d
EK1+SARS-HR1P, Tm=53.8 °C
EK1C4+SARS-HR1P, Tm=76.0 °C
[θ] (10-3 deg cm2 dmol-1)
Temperature (°C)
e
MERS-HR1P
EK1
EK1C4
EK1+MERS-HR1P
EK1C4+MERS-HR1P
[θ] (10-3 deg cm2 dmol-1)
Wavelength (nm)
f
EK1+MERS-HR1P, Tm=96.5 °C
EK1C4+MERS-HR1P, Tm >100 °C
[θ] (10-3 deg cm2 dmol-1)
Temperature (°C)

Supplementary information, Fig. S4 Biophysical characterization of EK1C4 and its complexes with HR1Ps. **a**. Circular-dichroism (CD) spectra for EK1, EK1C4, SARS-CoV-2-HR1P and their complexes in PBS. **b**. Melting curves of the complexes of EK1 / SARS-CoV-2-HR1P and EK1C4/SARS-CoV-2-HR1P. **c**. Circular-dichroism (CD) spectra for EK1, EK1C4, SARS-HR1P and their complexes in PBS. **d**. Melting curves of the complexes of EK1/SARS-HR1P and EK1C4/SARS-HR1P. **e**. Circular-dichroism (CD) spectra for EK1, EK1C4, MERS-HR1P and their complexes in PBS. **f**. Melting curves of the complexes of EK1/MERS-HR1P and EK1C4/MERS-HR1P.
