# Supplementary material for: Inhibition of SARS-CoV-2 (previously 2019-nCoV) infection by a highly potent pan-coronavirus fusion inhibitor targeting its spike protein that harbors a high capacity to mediate membrane fusion
Source: Cell Res. 2020 Mar 30;30(4):343–55. doi: 10.1038/s41422-020-0305-x (PMC7104723; doi:10.1038/s41422-020-0305-x)
Supplement: Supplementary file 5 — Supplementary information, Fig. S5 [file 41422_2020_305_MOESM5_ESM.pdf]

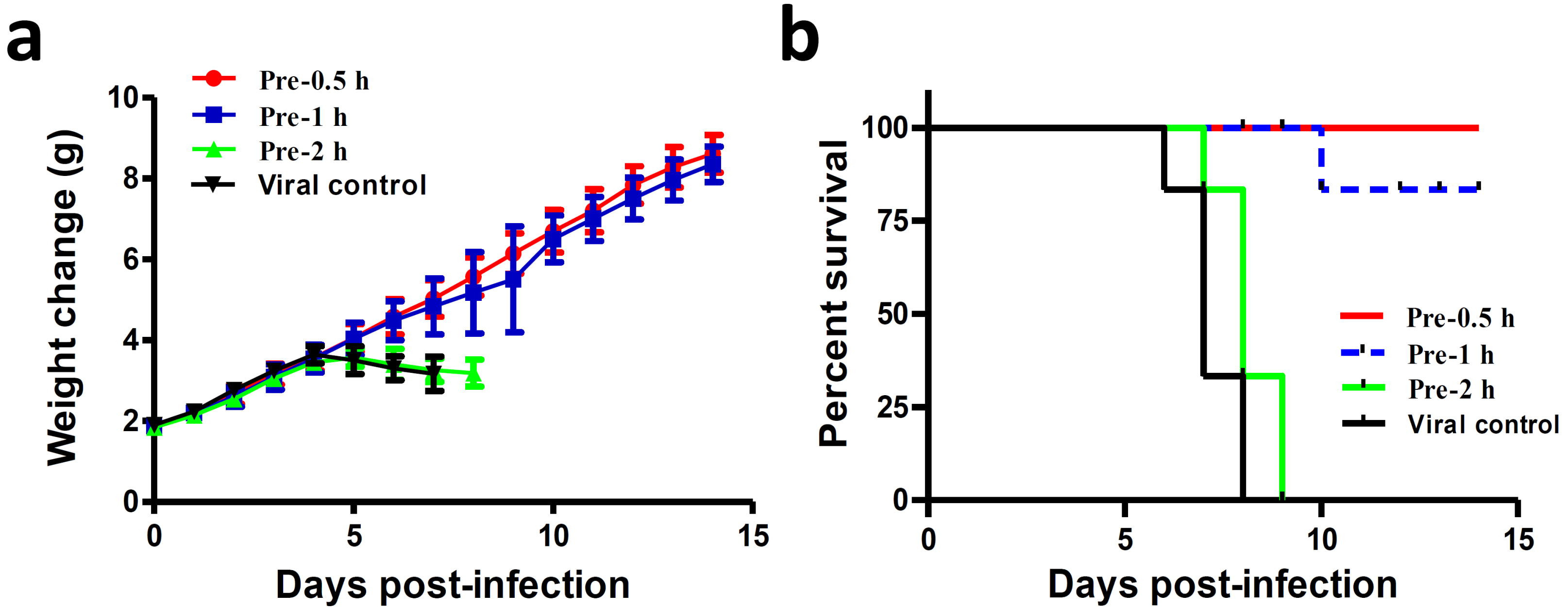

a
Pre-0.5 h
Pre-1 h
Pre-2 h
Viral control
Weight change (g)
0
2
4
6
8
10
0
5
10
15
Days post-infection
b
Percent survival
0
25
50
75
100
0
5
10
15
Days post-infection
Pre-0.5 h
Pre-1 h
Pre-2 h
Viral control

Supplementary information, Fig. S5 *In vivo* prophylactic efficacy of EK1 against HCoV-OC43 infection in mice. a. Body weight change of mice challenged with HCoV-OC43. **b**. Survival curves of mice challenged with HCoV-OC43.
