# Supplementary material for: Inhibition of SARS-CoV-2 (previously 2019-nCoV) infection by a highly potent pan-coronavirus fusion inhibitor targeting its spike protein that harbors a high capacity to mediate membrane fusion
Source: Cell Res. 2020 Mar 30;30(4):343–55. doi: 10.1038/s41422-020-0305-x (PMC7104723; doi:10.1038/s41422-020-0305-x)
Supplement: Supplementary file 6 — Supplementary information, Fig. S6 [file 41422_2020_305_MOESM6_ESM.pdf]

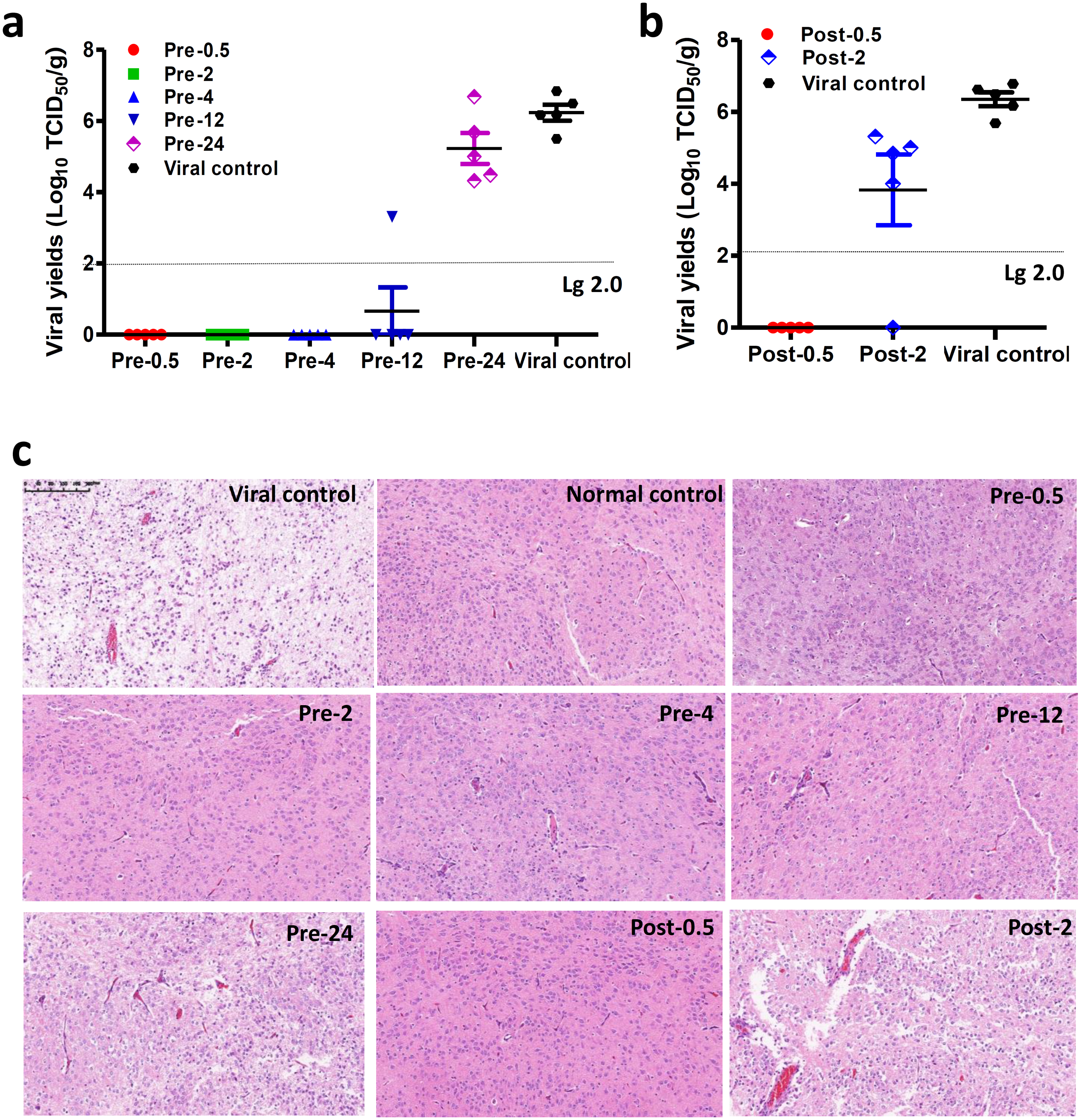
a
Viral yields (Log10 TCID50/g)
8
6
4
2
0
Pre-0.5
Pre-2
Pre-4
Pre-12
Pre-24
Viral control
Lg 2.0
b
Post-0.5
Post-2
Viral control
Lg 2.0
c
Viral control
Normal control
Pre-0.5
Pre-2
Pre-4
Pre-12
Pre-24
Post-0.5
Post-2

Supplementary information, Fig. S6 The prophylactic and therapeutic effect of EK1C4 administered into mice before and after viral challenge, respectively. a. Viral titer in brain tissues of mice in the viral control group and EK1C4-pre-treatment groups. The limit of detection is lg2.0 $TCID_{50}$/g. **b**. Viral titer in brain tissues in EK1C4-post-treatment groups. The limit of detection is lg2.0 $TCID_{50}$/g. **c**. Histological examination of mouse brains.
