# Supplementary material for: Inhibition of SARS-CoV-2 (previously 2019-nCoV) infection by a highly potent pan-coronavirus fusion inhibitor targeting its spike protein that harbors a high capacity to mediate membrane fusion
Source: Cell Res. 2020 Mar 30;30(4):343–55. doi: 10.1038/s41422-020-0305-x (PMC7104723; doi:10.1038/s41422-020-0305-x)
Supplement: Supplementary file 7 — Supplementary information, Fig. S7 [file 41422_2020_305_MOESM7_ESM.pdf]

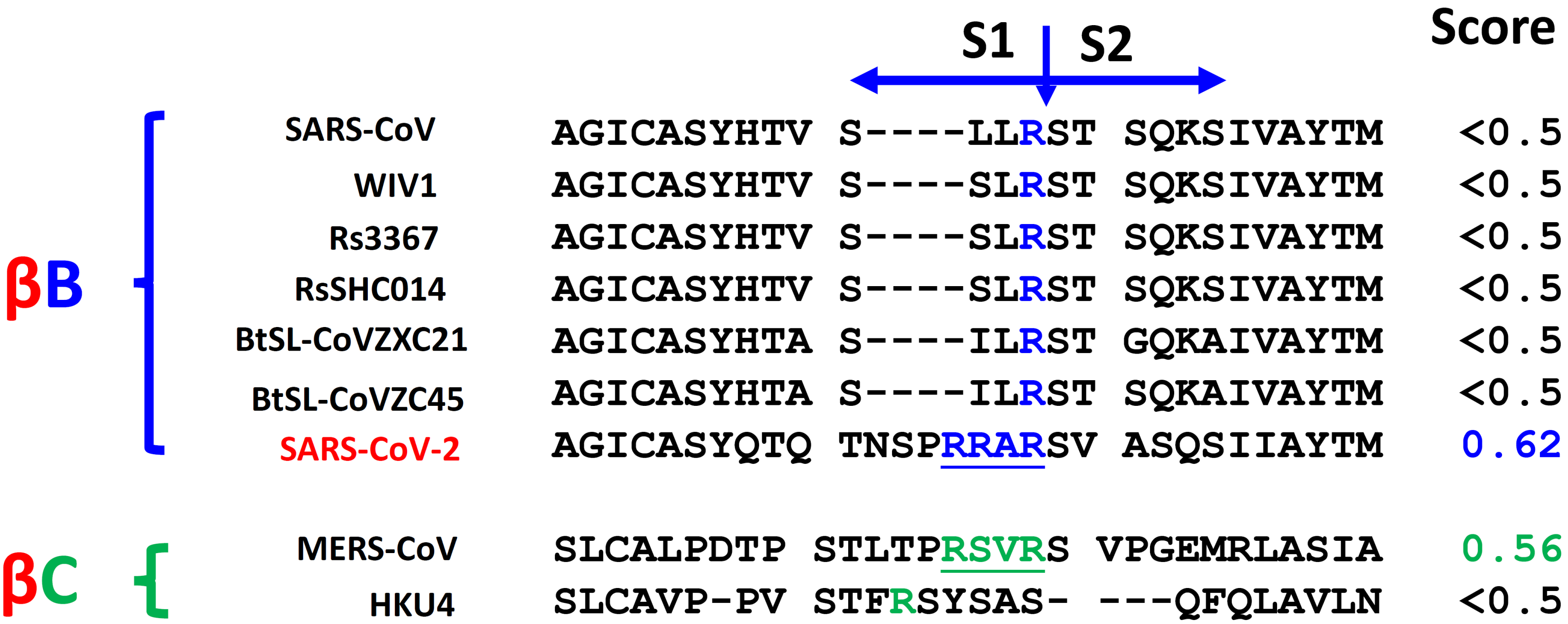
S1
S2
Score
βB
SARS-CoV AGICASYHTV S----LLRST SQKSIVAYTM <0.5
WIV1 AGICASYHTV S----SLRST SQKSIVAYTM <0.5
Rs3367 AGICASYHTV S----SLRST SQKSIVAYTM <0.5
RsSHC014 AGICASYHTV S----SLRST SQKSIVAYTM <0.5
BtSL-CoVZXC21 AGICASYHTA S----ILRST GQKAIVAYTM <0.5
BtSL-CoVZC45 AGICASYHTA S----ILRST SQKAIVAYTM <0.5
SARS-CoV-2 AGICASYQTQ TNSPRRARSV ASQSIIAYTM 0.62
βC
MERS-CoV SLCALPDTP STLTPRSVRS VPGEMRLASIA 0.56
HKU4 SLCAVP-PV STFRSYSAS- ---QFQLAVLN <0.5

Supplementary information, Fig. S7 Analysis of the S1/S2 furin-recognizable site in β-B and β-C coronaviruses.
