# Supplementary material for: Inhibition of SARS-CoV-2 (previously 2019-nCoV) infection by a highly potent pan-coronavirus fusion inhibitor targeting its spike protein that harbors a high capacity to mediate membrane fusion
Source: Cell Res. 2020 Mar 30;30(4):343–55. doi: 10.1038/s41422-020-0305-x (PMC7104723; doi:10.1038/s41422-020-0305-x)
Supplement: Supplementary file 8 — Supplementary information, Fig. S8 [file 41422_2020_305_MOESM8_ESM.pdf]

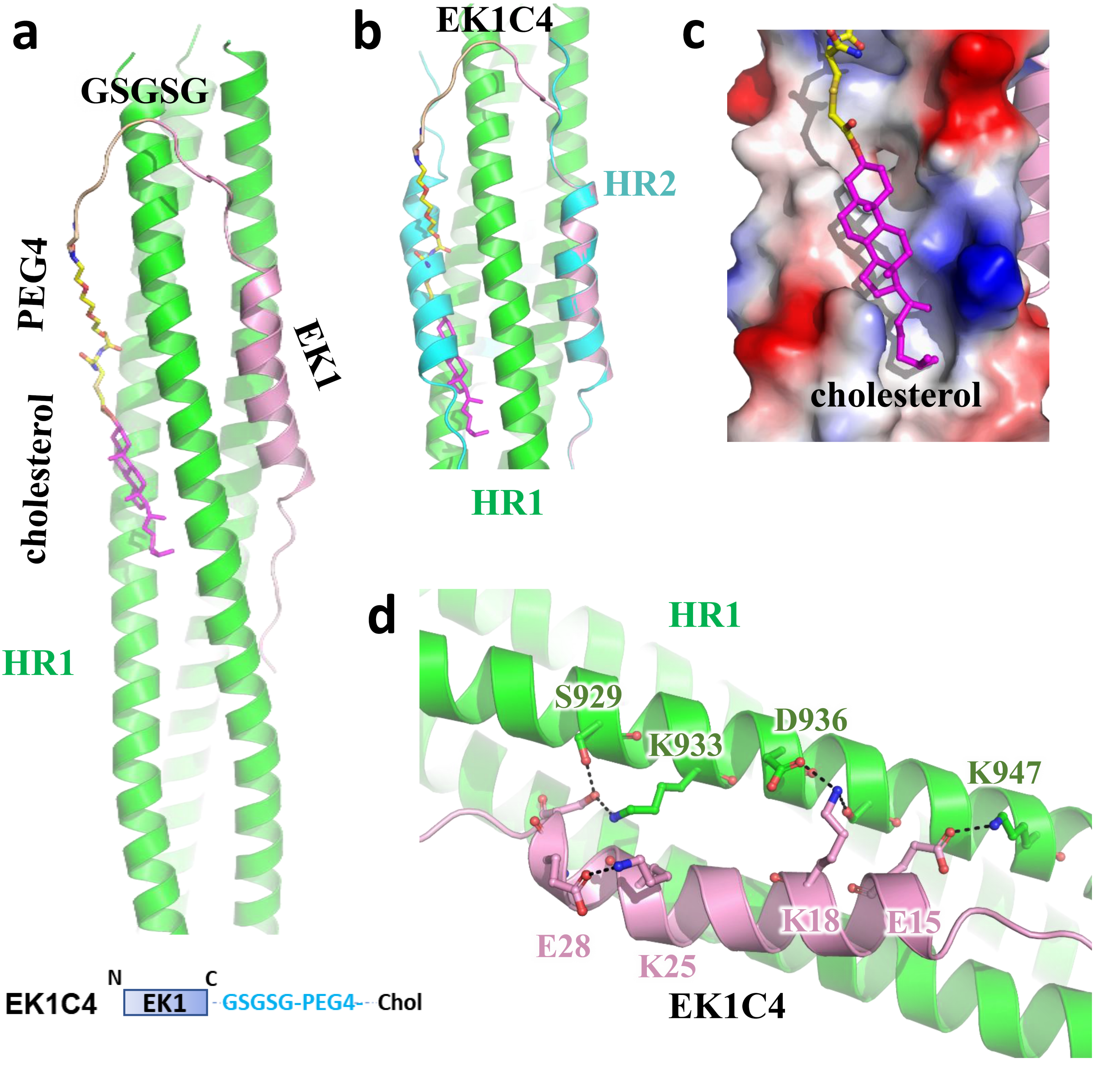
a
GSGSG
PEG4
cholesterol
EK1
HR1
b
EK1C4
HR2
HR1
c
cholesterol
d
HR1
S929
K933
D936
K947
E28
K25
K18
E15
EK1C4
EK1C4
N
C
EK1
GSGSG-PEG4-
Chol

Supplementary information, Fig. S8 The predicted model of interactions between EK1C4 peptide and HR1 domains of SARS-CoV-2. a. The interaction model of HR1 domain of SARS-CoV-2 to EK1 peptide and cholesterol group are predicted by SWISS-MODEL sever using 6XLT as reference, and Autodock 4 software, respectively. Structures are shown in cartoon representation, and each region of EK1C4 peptides is colored differently and labeled. **b**. The superposed structure of EK1C4 (Pink) and HR2 (Blue) peptides. **c**. The cholesterol domain is predicted to be buried in the hydrophobic grooves of SARS-CoV-2 HR1 domains. **d**. The EK1C4 peptide are predicted to interact with SARS-CoV-2 HR1 domain through several hydrogen bonds and salt bridges.
