# Supplementary material for: Inhibition of SARS-CoV-2 (previously 2019-nCoV) infection by a highly potent pan-coronavirus fusion inhibitor targeting its spike protein that harbors a high capacity to mediate membrane fusion
Source: Cell Res. 2020 Mar 30;30(4):343–55. doi: 10.1038/s41422-020-0305-x (PMC7104723; doi:10.1038/s41422-020-0305-x)
Supplement: Supplementary file 9 — Supplementary information, Fig. S9 [file 41422_2020_305_MOESM9_ESM.pdf]

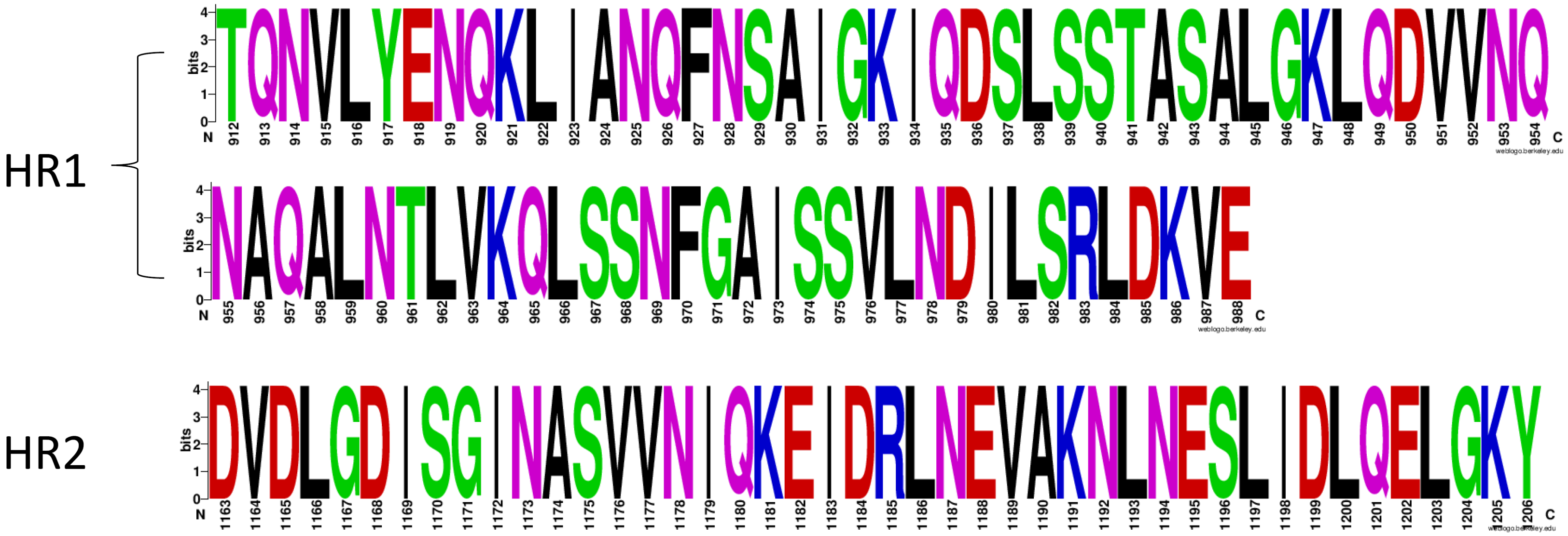
HR1
HR2

Supplementary information, Fig. S9 The identical sequence of HR1 and HR2 domains in 103 SARS-CoV-2 genomes.
